# Health technology assessment of public health interventions: an analysis of characteristics and comparison of methods—study protocol

**DOI:** 10.1186/s13643-018-0743-4

**Published:** 2018-05-23

**Authors:** Tim Mathes, Gerald Willms, Stephanie Polus, Constance Stegbauer, Melanie Messer, Corinna Klingler, Heidi Ehrenreich, Dea Niebuhr, Georg Marckmann, Ansgar Gerhardus, Dawid Pieper

**Affiliations:** 1 Institute for Research in Operative Medicine, Witten/Herdecke University, Ostmerheimer Str. 200, 51109 Cologne, Germany; 2 AQUA Institute for Applied Quality Improvement and Research in Health Care, Maschmuehlenweg 8-10, 37073 Goettingen, Germany; 3 Faculty of Health Science, Bielefeld University, Universitätsstr. 25, 33615 Bielefeld, Germany; 4 Institute of Ethics, History and Theory of Medicine, LMU Munich, Lessingstr. 2, 80689 Munich, Germany; 5 AOK-Bundesverband, Rosenthaler Str. 31, 10178 Berlin, Germany; 6 Department of Nursing and Health Sciences, University of Applied Sciences, Leipziger Straße 123, 36037 Fulda, Germany; 7 Department for Health Services Research, Institute for Public Health and Nursing Research, University of Bremen, Grazer Str. 4, D-28359 Bremen, Germany; 8 Health Sciences Bremen, University of Bremen, Bremen, Germany

**Keywords:** Public health, Health promotion, Health technology assessment, Methodology review

## Abstract

**Background:**

Conducting a health technology assessment (HTA) of public health interventions (PHIs) poses some challenges. PHIs are often complex interventions, which affect the number and degree of interactions of the aspects to be assessed. Randomized controlled trials on PHIs are rare as they are difficult to conduct because of ethical or feasibility issues.

The aim of this study is to provide an overview of the methodological characteristics and to compare the applied assessment methods in HTAs on PHIs.

**Methods:**

We will systematically search HTA agencies for HTAs on PHIs published between 2012 and 2016. We will identify the HTAs by screening the webpages of members of international HTA organizations. One reviewer will screen the list of HTAs on the webpages of members of international HTA organization, and a second review will double-check the excluded records. For this methodological review, we define a PHI as a population-based intervention on health promotion or for primary prevention of chronic or non-chronic diseases. Only full HTA reports will be included. At maximum, we will include a sample of 100 HTAs. In the case that we identify more than 100 relevant HTAs, we will perform a random selection. We will extract data on effectiveness, safety and economic as well as on social, cultural, ethical and legal aspects in a priori piloted standardized tables. We will not assess the risk of bias as we focus on exploring methodological features. Data extraction will be performed by one reviewer and verified by a second. We will synthesize data using tables and in a structured narrative way.

**Discussion:**

Our analysis will provide a comprehensive and current overview of methods applied in HTAs on PHIs. We will discuss approaches that may be promising to overcome the challenges of evaluating PHIs.

**Electronic supplementary material:**

The online version of this article (10.1186/s13643-018-0743-4) contains supplementary material, which is available to authorized users.

## Background

Health technology assessment (HTA) has become an important tool to support health policy decisions in many countries [1]. The majority of HTAs still focus on clinical medicine, in particular on pharmaceuticals. HTAs of public health interventions (PHIs) are still rare [2]. A 2010 survey conducted in five countries found that three in four HTAs covered a clinical topic, but only 5% of HTAs were on a public health issue [3].

HTA of PHIs poses some challenges [4]. PHIs tend to be highly complex due to varying intervention components (e.g. educational, psychological, or sociological), participants, contextual factors and multiple causal pathways [5]. Randomized controlled trials (RCTs) are often the most important data source for effectiveness in HTAs. However, RCTs on PHIs are often difficult to conduct because of ethical or feasibility issues. This leads to a lack of RCTs on PHIs [6]. HTAs on PHIs often have to rely on other study designs, in particular quasi-experimental designs such as controlled before-after studies or interrupted time series that are more prone to cofounding than RCTs. Because of these challenges, the standard tools for HTA (e.g. electronic databases, risk of bias assessment) are often not fit for purpose and may not even be applicable at all for PHIs [7, 8].

HTA agencies prepare HTAs on PHIs and consequently have to deal with these challenges. Only few HTA agencies have standardized, formalized methods specific for HTAs on PHIs, and the few suggested approaches are heterogeneous [8].

There is no systematic analysis on the methods applied in HTAs on PHIs. The aim of this study is to provide an overview of the methodological characteristics and to compare the assessment methods that have been applied in HTAs of PHIs. More specifically, our purpose is to get deeper insights into the approaches that are actually applied to deal with the challenges arising in HTAs of PHIs.

## Methods/design

We used the PRISMA-P checklist [9] for guiding the preparation of this protocol. Not all items of the checklist were applicable due to the nature of this work (see Additional file 1).

As no outcome of direct patient or clinical relevance is assessed in this work, the protocol could not be registered in PROSPERO.

### Searches

We will systematically search the webpages of the international umbrella organizations for systematic reviews and national HTA agencies. We will identify the HTA agencies by using the member lists of the following HTA umbrella organizations: the International Network of Agencies for Health Technology Assessment (INAHTA), Health Technology Assessment International (HTAi) and the European Network for Health Technology Assessment (EUnetHTA; 06/2016: 115 organizations). Because PHIs are very diverse, numerous search terms can be potentially relevant. Therefore, we will not use search terms for searching the webpages but screen the full lists of all published HTAs. One reviewer will perform the searches. References will be managed with EndNote X7.

### Inclusion criteria

Two reviewers will independently screen potentially relevant reports according to the following inclusion criteria:

1. Full HTA report as defined by INAHTA (see details below) [10]
2. Assessment of a public health intervention
3. Publication date: 2012 to 2016
4. Language: English, German, Spanish and French

We will apply the following key aspects of the INAHTA definition of full HTA reports for study selection [10]:

- ○ *Always*
  • Evaluate safety and effectiveness issues.
  • Determine the cost-effectiveness of the technology, e.g. through economic modelling (when it is appropriate).
- ○ *Always* conduct a comprehensive systematic literature review or a systematic review of high-level evidence.
- ○ *Always* critically appraise the quality of the evidence base.
- ○ *Optionally* address ethical, social and legal considerations.

We will exclude all literature review-based HTAs using accelerated (e.g. rapid reviews) or abbreviated HTA/systematic review methods, overviews of reviews (or umbrella reviews), scoping reviews, mini-HTAs, etc. [10, 11]. We will exclude protocols for HTAs because we are primarily interested in the actual application of methods for dealing with the challenges when evaluating a PHI.

Public health is a broad discipline and not homogeneously defined in the literature. In this review, we will consider only population-based interventions on health promotion and interventions for primary prevention of chronic (e.g. cardiovascular diseases, diabetes and non-communicable [e.g. injury]) or infectious disease to ensure consistent study selection [12]. We will exclude HTAs on screening and vaccination because these require special evaluation methods (e.g. diagnostic accuracy studies, modelling) [13].

As our intention is to provide an overview of the currently applied approaches, we will include only reports published in the last 5 years (2012–2016). In case that a HTA agency published more than ten relevant HTAs within the past 5 years, we will randomly select ten reports of this agency to avoid overrepresentation of one HTA agency. We will include a maximum of 100 HTAs in the analysis. In the case that this threshold is exceeded, we will adapt the maximum number of HTAs per agency accordingly. We will include at least one report for each agency, even when the threshold of 100 HTAs will be exceeded.

One reviewer will screen all abstracts and full texts, and a second reviewer will screen the excluded records only (liberal acceleration).

A flow diagram will illustrate the selection process, and we will provide a list of the excluded HTA reports to ensure transparency of the selection process.

### Data extraction

We will prepare standardized tables for data extraction. We will pilot the data extraction tables before the conduction of the review with HTAs published before 2012. Two reviewers will independently extract the data.

For all HTA reports, we will extract data on (i) population and (ii) intervention characteristics, (iii) country of origin, (iv) review planning, (v) definition of the research question and (vi) the development of the theoretical framework/theory of change.

Furthermore, we will extract detailed information on the applied methods for each domain (effectiveness, safety and economic as well as on social, cultural, ethical and legal aspects).

#### Health (effectiveness/safety) aspects

If the assessment is based on a systematic review, then we will extract data on the following issues:

- Included study designs (e.g. interrupted time series)
- Search strategy (electronic databases and additional searches)
- Data extraction (items, method, involved reviewers)
- Risk of bias/quality assessment (tool, method, involved reviewers)
- Considering context/setting in the assessment
- Assessment of applicability/generalizability (tool, method, involved reviewers)
- Assessment of intervention integrity
- Assessment of sustainability
- Assessment of heterogeneity
- Integration qualitative and quantitative studies
- Information on subgroups (see below)

Many different primary study types (e.g. surveys) or synthesis methods (e.g. rapid reviews) can be used in addition to full systematic reviews to assess the effectiveness of (complex) health interventions [14–16]. We will also extract data on features of other synthesis methods than “traditional” systematic reviews (e.g. realist reviews) and on other assessment methods such as trials, surveys and expert reviews.

#### Economic aspects

Also, the economic evaluation of PHIs presents some additional methodological challenges. Methodological challenges refer to the complexity of the intervention, the choice of comparators and the consideration of multiple or intermediate outcomes. These issues often require complex modelling techniques.

In the economic domain, we will consider whether a primary economic evaluation and/or a systematic literature search of existing economic evaluations is performed.

For primary economic evaluations, we will extract the following information:

- Type of economic evaluation
- Choice of comparators
- Time/analysis horizon
- Perspective of analysis
- Costs considered (e.g. direct medical costs, indirect costs)
- Sources/measurement of costs
- Valuating resources (pricing)
- Sources/measurement of outcomes
- Valuation of outcomes (e.g. time-trade-off)
- Discounting
- Modelling approach (e.g. Markov model, system dynamics)
- Sensitivity analysis
- Presentation of results

For systematic reviews of economic evaluations, we will extract:

- Scope of the systematic review (exclusively a systematic review of economic evaluations or systematic review to inform primary economic evaluation)
- Included economic evaluation types
- Literature search strategy (databases, additional sources, involved reviewers)
- Data extraction (items, method)
- Assessment of methodological quality (tool, method)
- Assessment of generalizability/transferability/applicability (tools, procedure)
- Presentation of cost data (e.g. currency conversion)
- Method for data synthesis (narrative, graphical, meta-analysis)

Furthermore, we will consider if the results of the economic evaluation can explicitly influence the final decision (e.g. through a cost-effectiveness threshold).

We will extract whether the HTA includes a budged impact analysis but no details on the methods for the budget impact analysis.

#### Social and cultural aspects

If social and cultural aspects are considered, then we will extract information on [17]:

- The social and cultural aspects addressed
- Theoretical framework chosen
- Assessment method chosen
  - Quantitative
  - Qualitative
  - Mixed methods
  - Literature review of qualitative and/or quantitative studies
- Explicit implications for the HTA

#### Legal aspects

Information on the approaches for considering legal aspects will be extracted. We will consider the following legal aspects [15]:

- Informed consent
- Alternative forms of consent
- Privacy and data protection
- Market authorization of medical devices and medicinal products
- Clinical trials
- Intellectual properties
- Reimbursement in public health care systems
- Special medical fields

There is no standardized approach for incorporating legal aspects [18]. Therefore, we will further specify the data extraction for legal aspects after the literature screening.

#### Ethical aspects

If possible, we will extract information on:

- The ethical issues addressed
- The normative framework chosen
- The methods employed for arriving at recommendations
- Explicit implications for the HTA

As this part of systematic reviews is largely underdeveloped and no clear standards are formulated, we are not able to predict what approaches were chosen [19]. We expect considerable variation in the understanding of ethical aspects and the methods employed. Therefore, it might become necessary to adapt this list after a first screening of the literature (but before in-depth analysis of the publications identified).

### Risk of bias assessment/quality

We will not assess the quality of HTA reports because we focus on exploring methodological features of HTAs on PHIs. We will include only HTAs that perform a systematic literature search and perform a critical appraisal of included studies, which can be considered as minimal requirements for HTA quality.

### Data synthesis

We will tabulate data for each domain. The information will be synthesized in a structured narrative way for the respective domain and also across domains.

### Statistics

We will describe dichotomous and nominal variables using absolute numbers and percentages. We will prepare means and standard deviations for metric variables. For ordinal and skewed metric variables, we will calculate median and ranges.

All analyses will be performed using SPSS 23 software.

### Analysis of subgroups

We plan to perform two subgroup analyses. First, we will consider HTAs on health promotion separately from HTAs on primary prevention. Second, HTAs will be analysed according to the different health care systems (e.g. Beveridge model, Bismarck model, National Health Insurance model), target audiences (e.g. physicians, policy decision makers) and evaluation level (e.g. hospital/primary care, region, national) to identify justified methodological differences (e.g. perspective of analysis, assessment of applicability). We will code the subgroups based on description of the intervention, country of origin, setting and perspective of the analysis.

## Discussion

HTA is a tool to support policy decisions [20]. A comprehensive and transparent assessment is in particular important in the field of public health because the decision process often involves a large variety of stakeholders.

The analysis will provide a comprehensive overview of methods applied in HTAs on PHIs. We will get insights into promising approaches for dealing with the challenges when evaluating PHIs. Furthermore, our work might identify important research gaps. This analysis can stipulate further development and harmonization of methods for HTAs on PHIs. An advancement of the methods can contribute to a higher degree of acceptance of HTAs on PHIs by policy makers and improved policy decision-making based on the principles of evidence-based medicine.

We are aware that especially in the evaluation of a PHI, social and ethical considerations should be addressed. Social and cultural norms influence how people perceive a health issue and accept an intervention and its implementation [15]. These might cause differences in effectiveness between groups. To avoid the exclusion of a majority of HTAs, we decided to include also HTAs evaluating only effectiveness/safety and economic aspects.

Our work has some limitations. First, our focus on health promotion and interventions for primary prevention, as public health covers a broader field encompassing environmental, behavioural and clinical interventions for secondary and tertiary prevention. This decision was made because we want a sample of HTAs that represents the specific characteristics of PHIs and allows unambiguous differentiation from other medical fields. While we have not found another satisfying way that clearly distinguishes PHIs from other interventions, we presume that our results are also applicable to other PHIs. Second, the results of our analysis might be biased toward European and North American countries because of our language restrictions. A third limitation is that we will not arrive at a representative sample of HTAs because of our strict inclusion criteria (full HTAs, only literature-based HTAs). This decision was made because we deduced our inclusion criteria from a previous work that analysed the methods in manuals for HTAs on PHIs [8]. Thus, this work will provide only an extract of “state of the art” methods. We acknowledge that these methods are not always feasible because of lack of resources to perform an in-depth analysis or lack of appropriate literature.

In a future project, we will widen our scope and also consider HTAs that only use accelerate review methods (e.g. rapid reviews) or primary data analysis (e.g. quasi-experimental designs, expert surveys).

## Additional file

Additional file 1: PRISMA-P+checklist. (DOCX 39 kb)

## Abbreviations

EUnetHTA: European Network for Health Technology Assessment
HTA: Health technology assessment
INAHTA: International Network of Agencies for Health Technology Assessment
HTAi: Health Technology Assessment International
PHI: Public health intervention
RCT: Randomized controlled trial
